# Quantitative Determination of Technological Improvement from Patent Data

**DOI:** 10.1371/journal.pone.0121635

**Published:** 2015-04-15

**Authors:** Christopher L. Benson, Christopher L. Magee

**Affiliations:** Department of Mechanical Engineering, Massachusetts Institute of Technology, Cambridge, Massachusetts, United States of America; Nagasaki University, JAPAN

## Abstract

The results in this paper establish that information contained in patents in a technological domain is strongly correlated with the rate of technological progress in that domain. The importance of patents in a domain, the recency of patents in a domain and the immediacy of patents in a domain are all strongly correlated with increases in the rate of performance improvement in the domain of interest. A patent metric that combines both importance and immediacy is not only highly correlated (r = 0.76, p = 2.6*10^-6^) with the performance improvement rate but the correlation is also very robust to domain selection and appears to have good predictive power for more than ten years into the future. Linear regressions with all three causal concepts indicate realistic value in practical use to estimate the important performance improvement rate of a technological domain.

## Introduction

It is possible to quantify the improvement of a technological domain over time, as was first introduced by Moore [1] and has since been explored more broadly and deeply by many others [2–9]. All of these authors find exponential relationships between performance and time or equivalently that the fractional (or percentage) change per year is constant. Specifically, if q is performance at time t and q_0_ performance at a reference time, t_0_,

$$q=q_{0}\;\text{exp}\left(k\left(t-t_{0}\right)\right)$$ (1)

The exponential constant (k) is referred to here as the technological improvement rate, which represents the performance improvement over time for a specific generic function that the technological domain is accomplishing. Estimates for k are determined by first constructing a functional performance metric (FPM) that is a measure of the generic function for a technological domain and includes the factors that affect the purchasing decision for artifacts embodying the technology (for example: Watts/$ for Solar PV). Next, data points that measure the FPM are collected over a range of time: a technological improvement rate is determined by an exponential regression vs. time and is statistically analyzed to examine robustness and reliability. While there has been considerable research into finding these improvement rates for different technologies and understanding the best way to measure them [4,5], there has been relatively little work done to understand why there may be differences in improvement rates among technologies.

One of the sources of data that has been widely used for understanding technological change in recent years is patent data [10–15]. Patents are an attractive choice for analyzing technological change because they are: generalizable, objective, quantitative and qualitative. Patents include many technical fields over a long period of time, and thus allow for easier generalization of the research. There are specific criteria for an invention to be patented, which creates an objective standard as to what counts as an invention (as opposed to a subjective list of innovations in a field). Each patent is well tracked and includes a wealth of meta-data, and thus allows for quantitative analysis.

While many aspects of patents make it an attractive data source for innovation analyses, patents are limited in that they may not cover all inventions or discoveries due to specific patentability criteria that makes it impossible to patent some things (such as Maxwell’s equations) and not all inventions are patented for both economic (secrecy) and competitive reasons (i.e. universities could not collect royalties on patents before 1980). Additionally, the temporal nature of patents can lead to truncation issues with patent data as has been explored by [16–18]. An important aspect of the present work is testing the severity of these shortcomings relative to technological progress in a fairly wide set of technological domains—see discussion relative to Hypothesis 0 throughout the paper.

### Literature Review and Development of Hypotheses

Although there is no existing theory that directly attempts to explain the differences between technological improvement rates in technological domains, there are a large number of useful theoretical writings on technological change. This section reviews the technical change literature in order to build upon prior work in Benson and Magee [19] to establish hypotheses that are testable from patent data. Since the quantitative basis for this study is linking the technological improvement rates with patent characteristics, we are (at least implicitly) making a foundational assumption. The critical assumption is that patents indeed capture enough information that is relevant to technological progress to achieve significant correlations between patent characteristics in domains and the rate of progress in the same domains. If patents do not sufficiently contain the important information distinguishing technical progress in a variety of domains, this assumption is problematic. The assumption can be represented by the following hypothesis.

HYPOTHESIS 0: *The differences in technological improvement rates among technological domains can be accounted for by the differences among patent characteristics of the domains*.

The remainder of this section develops hypotheses based upon various concepts from the literature on technological change. The concepts are operationalized by relationships to specific patent characteristics and the concepts and patent characteristics are summarized in Table 1. Each of these patent metrics is treated as an independent variable with the k-value as the dependent variable whose variation across domains we will test for each hypothesis. However, the structure followed in the reasoning is that the concepts are what cause both variation in the performance improvement rate (k) and the patent metric. The equations and specific manner of measuring the patent characteristics will be discussed later in the Data and Methods section.

**Table 1:** Description of Independent Variables.

****Patent characteristics****	****Concept****	****Description****
(1) Simple Patent Count	A: Effort	number of issued US patents in a domain from 1976–2013
(2) Average number of forward citations	B: Importance of Patents	average number of times each patent in a domain is cited
(3) Ratio of important patents	B: Importance of Patents	ratio of patents with cited by over 20 to total patents in a domain
(4) NPL Ratio	C: Impact of Science	ratio of scientific citations to total citations from the domain patents
(5) Average publication year	D: Recency	the average date of publication for all patents in a domain
(6) Average Age of backward citation	E: Immediacy	average age of backward citations for each patent (averaged over the domain) at the time of the citing patents publication
(7) Price Index (3 years)	E: Immediacy	average proportion of citations that a domain patent receives within 3 years of publication
(8) Ratio of Backward Citations to Other Domains	F: Breadth of Knowledge	ratio of citations from patents in the domain to patents in other domains
(9) Mean publication date of backward citations	D & E: Recency and Immediacy	average date of publication for backward citations from patents in a domain
(10) Average City by within 3 years	B & E: Immediate Importance	average number of citations that a domain patent receives within 3 years of publication

#### Concept A: Effort in a Domain

There are several aspects of technological evolution where the demand or usage could play an important role in the relative rate of improvement in a technological domain. Wright's [20] well-known paper related the cumulative production of a product with decreasing costs. Arrow, in his important 1962 paper [21], named this effect “learning by doing” and developed a model that showed that more highly used technologies would enable more opportunity to 'learn by doing' in production. Although Wright’s and other early efforts [22,23] focused on production of a given design in a given factory, later the concept was generalized so that cumulative production serves as a proxy for effort of any kind [4,24]. In this generalization, cumulative production is summed over the domain (or industry) of interest. The generalization is consistent with revenue and R&D spending increasing with production volume [25].

A direct relationship between R&D effort and technical improvement has been discussed by many researchers of technical change. Christensen [26] related the technical improvement of areal density of hard disks to the increase in engineering effort, and Foster [27] considered R&D effort the major variable in determining improvement. A relationship between R&D effort and the number of patents produced in a particular domain is supported in the work of Margolis and Kammen [28]. Thus, our study uses patent output to test the concept that more inventive effort presumably by more R&D spending (measured by patent output) results in increases in technological performance improvement. As a result, the first hypothesis is:

HYPOTHESIS 1: *The performance improvement rate in technological domains should be higher in domains with increased number of patents within that technological domain*

#### Concept B: Importance of patents in a domain

One of the main explanations of technological change in the literature is based upon categorizing the improvements or inventions within a technology into distinct categories. Many researchers [29–31] argue the significance (perhaps even dominance) of a small set of very important inventions in technological change. In almost all cases of the innovation categorization concept, there is both a lesser and a greater classification. For example, incremental innovation achieves small changes, while radical innovation results in much more change. Similar differentiation can be made for component vs architecture and “normal” vs breakthrough while punctuated and disruptive changes are also large. Sood and Tellis [32] have noted that many of these terms are *'intrinsically problematic because they define an innovation in terms of its effects rather than its attributes'*. For our study, the impact of this concept is that we assume that technological change is faster for domains with more important inventions. Thus, we attempt to characterize the importance of innovations in different domains.

The use of forward citations for estimating the importance of a single patent was first suggested on the basis of study of the economic impact of specific patents in a domain (Computed Tomography) relative to other patents in that domain [10,33]. It has been supported in a number of other studies [34,35] including one where patent citations are used to find rate-limiting components on computer improvements [36]. More recently, research results [37,38] have independently found significance for forward citations in value of patents from detailed statistical analysis and from actual patent auctions.

Hypothesis 2 seeks to assess the influence of the average importance of patents in a particular domain, with the intuition being that a domain with patents of higher average importance should improve more rapidly than those with lower average importance.

HYPOTHESIS 2: *Technological domains with a higher average number of citations to patents in the domain should have higher rates of improvement of performance*.

Hypothesis three involves the impact of particularly important inventions on technological improvement. It is reasonable that technological domains with a larger concentration of very important inventions would improve in performance faster than those with less concentration of such inventions.

HYPOTHESIS 3: *Technological domains with a higher frequency of patents that are cited a large number of time should have higher rates of improvement in performance*.

#### Concept C: The importance of science in a domain

Technology change researchers recognize an essential role for science in technological development; however the complexity of the specific mechanism has continued to unfold. Schumpeter’s early contribution [39] and Bush’s well-known paper [40] are often noted as early statements about the importance of science. The short-hand name for science leading to technology—the linear model- became a straw-man for oversimplification of technology development: Freeman [41] claimed that at one point in time it was nearly impossible to read an article related to technological change or related policies without discussing the linear model. Many missing elements were discussed [42–44]: Godin [45] describes how even Bush modified his connection between basic and applied research around 1960 to include the idea of development. At present, there is arguably an emerging consensus [46,47] that science and technology are intimately connected but that the interconnection is highly complex [48–52]. As one example supporting the idea that domains more closely related to science should improve faster, the results of Klevoric et al [48] indicate that “opportunities” are greater for domains that are more closely related to science (they note pharmaceuticals and chemicals as two examples) than are the opportunities available to domains that are not as closely linked to science (pumps and motors are two examples they give).

To test this idea through patent information, one must connect science directly to patents: some have used a patent characteristic which is the number of backward references to scientific papers [49] and others have used the fraction of backward references by a patent to the non-patent literature which are mostly citations to scientific articles [47,53–55]. For understanding differences in rates between domains, this concept suggests that *domains* whose patents cite more scientific articles will improve more rapidly than those who cite less such articles; the resulting hypothesis is:

HYPOTHESIS 4: *Technological domains with a higher frequency of citations to the scientific literature should have higher rates of improvement in performance*.

#### Concept D: Recency of work in (or emergence of) a domain

The basic intuition underlying concept D is the idea that more rapidly improving domains are newer. Schoenmakers and Duysters [56] showed that more important inventions tended to rely upon newer technologies and Nerkar’s [12] results indicate a positive impact of recency on the importance of pharmaceutical patents; however, application of recency to comparison among domains has not been previously considered. Thus, we examine whether domains that are newer improve at a more rapid pace than their older counterparts; the resulting hypothesis is:

HYPOTHESIS 5: *Technological domains whose patents are newer should have higher rates of improvement in performance*.

#### Concept E: Immediacy of utilization of domain patents and immediacy of knowledge utilized by domain patents

The relationship between more immediate science and more rapidly improving scientific fields provides a promising analogy for the importance of immediacy of patents in technological improvement. The connection between immediacy of science and higher scientific improvement rates was suggested by Price [57], who showed that fast improving scientific fields follow a 'research front' that relies mainly on very recently published papers. We should note that Price was not referring to how new a field was as discussed in concept D but instead at any time, how closely related the citations were to the time in question which we therefore label immediacy. Patents that are used more quickly indicate faster incorporation of new knowledge and we conjecture that more rapid incorporation of knowledge also results in more rapid improvement in performance.

HYPOTHESIS 6: *Domains whose patents are cited relatively more often earlier (as opposed to later) in their existence should have higher rates of improvement*

There are two ways immediacy can be important. One is the tendency for patents in a domain *to be cited* soon after issuance as captured in hypothesis 6: the second is for patents in a domain *to cite* more immediate patents. Since domains in a patent typically cite patents not in the domain ~90% of the time, these relationships (backward and forward citation immediacy) need not have the same effect. Thus, a further immediacy hypothesis is:

HYPOTHESIS 7: *Domains that cite more immediate patents should have higher rates of technological progress*

#### Concept F: Breadth of Knowledge

The breadth of knowledge concept reflects combining knowledge from different domains, assuming that the use of information from a larger variety of different sources is likely to result in improved technological outcomes. Rosenberg [58] showed that such “technological spillover” greatly impacted the quantity and quality of technological change in the United States in the 20th century—a result supported by others [59,60]. Indeed, a recent paper by Nemet and Johnson [61] state that one of the most fundamental concepts in innovation theory is that ‘important inventions involve the transfer of knowledge from one technical area to another”, a claim which is supported by many others [11,48,59,60,62].

Trajtenberg et al [47] studied knowledge breadth from patent data by considering the multiple patent classes for single patents and their results indicate that the technologies with broader technological roots enable more generalizable technologies. However, in similar studies (but with emphasis on backward citations) neither Nemet and Johnson [61] or Benson and Magee [63] found any impact of knowledge breadth on importance of patents within domains. Despite the lack of clarity of impact within a domain, we test an “extension” of this concept in this work: domains that rely upon knowledge from a broader knowledge base are likely to improve more quickly.

HYPOTHESIS 8: *Technological domains that cite higher fractions of patents from other domains will have higher rates of improvement*.

### Hybrid Concepts

#### Recent Immediacy

The concepts of recency and immediacy can work together to increase the technological improvement rate. The intuition is that the combination of two independently important drivers will lead to an even stronger effect on the rate of technological improvement through a single combined metric. A metric for recent immediacy that is tested in this paper is the average publication date of all backward citations by patents in a domain. This is directly equivalent to adding the positive linear effects of H5 (patent publication date) and H7 (backward citation age at time of patent publication)

HYPOTHESIS 9: *Technological domains whose patents on average cite patents that are newer will have higher rates of improvement*.

#### Immediate Importance

This hybrid concept combines immediacy and importance and thus argues that domains whose patents are more important in the *early* years of a patent’s existence are more dynamic. Although the concept has not previously been developed in the literature (to our knowledge), it is consistent (in a more continual way) with the disruption concepts of Christensen [64] and the discontinuity arguments of Anderson and Tushman [65] and others who support the importance of discontinuities. The specific hypothesis:

HYPOTHESIS 10: *Domains whose patents are highly cited in the early years of their existence should progress more rapidly*.

## Data and Methods

We attempt to explain the variation in k-values (the dependent variable) among domains by the variation in the various patent metrics (independent variables). The objective is to determine which of the patent metrics correlate significantly with the k’s.

There are three main components of the methodology. The first is selecting domains and finding their corresponding k values. For this study, we used the results for 28 domains that are covered in detail by Magee et al [5], these 28 domains represent over 10% of the US Patent Database and provide a sufficient sample size for generalization of results. The next major component is to locate a set of patents that represent each of the same technological domains so that the patent metrics listed earlier can be extracted from a representative set of patents. This process was done using the classification overlap method described in Benson and Magee [66] and later expanded [67,68]. This method takes advantage of the fact that many patents are classified in multiple International (IPCs) and/or US patent classes (UPCs) and uses the overlap of IPC and UPCs that are most closely related to a technology in order to clearly define a specific set of US issued patents to represent the technology of interest. For example, milling machine technologies are represented by the overlap of the US patent class 409 and the international patent class B23C. This set can then be downloaded easily from www.patsnap.com using the search term UCL:(409) AND ICL:(B23C), which is how all of the sets of patents were collected. Due to the fact that many patents are listed in multiple IPC and/or UPCs it is possible for a patent to represent multiple domains, for example, 1.4% of the ‘Integrated Circuit Processors’ patents are also represented in ‘Solar PV’ patent set. In the latter paper (68), Benson and Magee locate sets of patents that represent each of the 28 technological domains of interest.

In the third component of the methodology, the patent sets are analyzed to find the set of patent metrics for each technological domain and are then compared quantitatively with the k-value for each domain. The specifics of calculating the patent metrics for hypothesis testing are now discussed briefly below. The patent set characteristics and the k values for the 28 domains studied are given in Table A (in S1 File).

### Hypothesis 0

Hypothesis 0 is the most general and is tested by the ability of the patent data to explain the differences in technological improvement rates. This hypothesis can be supported by a patent metric that correlates highly with k and has statistical significance. The hypothesis is strongly reinforced by a set of patent metrics that correlate with k that all have statistical significance.

### Hypothesis 1

The *Simple Patent Count* is the total number of patents within a technological domain. In this research, this includes patents that were published between January 1st, 1976 and July 1st, 2013. This measure is calculated using Equation 1 where SPC is the simple patent count, t is the date, and Pt is the set of patents issued on that particular date, and ‘*COUNT()’* returns the total number of elements in a set.

$$SPC=\sum_{t=1/1/1976}^{7/1/2013}COUNT(P_{t})$$ (2)

Two patent metrics are used to test Concept B and both are directly related to the future (or forward) citations to the patents within a domain. These attempt to measure the impact that a field has on future inventions.

### Hypothesis 2

The *Average Number of Forward Citations per Patent* is the average number of Forward citations for the patents in a technological domain. This measure is calculated using Equation 3 where SPC is the simple patent count, and *FC*_*i*_ is the number of Forward citations for patent *i*.

$$\frac{\sum_{i=1}^{SPC}\sum_{j=1}^{FC_{i}}1}{SPC}$$ (3)

### Hypothesis 3

A test of Hypothesis 3 (high frequency of highly cited patents) is the *Total Number of Patents with more than 20 Forward Citations*. The specific cutoff of 20 citations is based on work done by Schoenmakers and Duysters [56]. This measure is calculated using Equation 4 where SPC is the simple patent count, *FC*_*i*_ is the number of Forward citations for patent *i*, and the function *IF(arg)* only counts the values if the argument is satisfied. In this situation, *IF(FC*_*i*_ *>20)* will only be counted if patent *i* has more than 20 forward citations.

$$\sum_{i=1}^{SPC}IF\left(FC_{i}>20\right)$$ (4)

### Hypothesis 4

The *Non-Patent Literature Citation Ratio* is the ratio of citations in a patent to non-patent literature (NPL)—usually scientific journals—to the total citations in the patent and for our purposes is averaged over all patents in the domain. This measure is calculated using Equation 5 where SPC is the simple patent count, *NPL*_*i*_ is the number of non-patent literature citations for each patent *i*, and *BC*_*i*_ is the number of backward patent citations for each patent *i*. Not all patents have citations to NPL; in our data sets, 43% of the patents cited at least 1 NPL reference, ranging from 16% of patents in the ‘electrical information transmission’ patent set to 93% of patents that represent ‘genome sequencing’ citing NPL.

$$\frac{\sum_{i=1}^{SPC}\frac{NPL_{i}}{NPL_{i}+BC_{i}}}{SPC}$$ (5)

### Hypothesis 5

Hypothesis 5 is evaluated using the *Average Publication Year* for the patents in a domain, which provides a simple and effective method of gauging the recency of a technological domain. In this research, this includes patents that were published between January 1st, 1976 and July 1st, 2013. This measure is calculated using Equation 6 where SPC is the simple patent count and $t_{i_{pub}}$ is the publication year of patent *i*.

$$\frac{\sum_{i=1}^{SPC}t_{i_{pub}}}{SPC}$$ (6)

### Hypothesis 6

Hypothesis 6 is tested by the *Price Index (3 years) [57]*. This metric is an immediacy metric for *usage of information generated* in a domain and thus involves forward citations. The measure is calculated using Equation 7 where SPC is the simple patent count, *FC*_*i*_ is the number of Forward citations for patent *i*, $t_{i_{pub}}$ is the publication year of patent *i*, $t_{ij_{pub}}$ is the publication date of forward citation *j* of patent *i*, and the function *IF(arg)* only counts the values if the argument is satisfied.

$$\frac{\sum_{i=1}^{SPC}\sum_{j=1}^{FC_{i}}IF(t_{ij_{pub}}-t_{i_{pub}}\leq 3)}{\frac{\sum_{i=1}^{SPC}\sum_{j=1}^{FC_{i}}1}{SPC}}$$ (7)

### Hypothesis 7

The immediacy concept is also tested by the *Average Age of Backward Citations*. This measure is calculated using Equation 8 where SPC is the simple patent count, *BC*_*i*_ is the number of backward citations for patent *i*, $t_{ji_{pub}}$ is the year of publication of backward citation *j* of patent *i* and $t_{i_{pub}}$ is the publication year of patent *i*. Note that this equation is the average publication date minus the average publication date of backward citations.

$$\frac{\sum_{i=1}^{SPC}t_{i_{pub}}}{SPC}-\frac{\sum_{i=1}^{SPC}\sum_{j=1}^{BC_{i}}t_{ji_{pub}}}{\sum_{i=1}^{SPC}\sum_{j=1}^{BC_{i}}1}$$ (8)

### Hypothesis 8

The patent metric that is used to evaluate Hypothesis 8 is the *Ratio of Backward Citations to other Domains*. This measure is calculated using Equation 9 where SPC is the simple patent count, and *BC*_*i*_ is the set of backward citations for patent *i, P_i_* is the total set of patents within the domain and ∪ is the union of two sets across all values of *i*, ∩ is the intersection between two sets and COUNT() counts the number of elements in a set.

$$1-\frac{COUNT(\overset{SPC}{\underset{i=1}{\cup }}P_{i}\cap BC_{i})}{SPC}$$ (9)

### Hypothesis 9

Combining the recency and immediacy concepts, it is possible to test a combination of the two using the *Average Date of Publication of Backward Citations*. This measure is calculated using Equation 10 where SPC is the simple patent count, *BC*_*i*_ is the number of backward citations for patent *i*, $t_{ji_{pub}}$ is the year of publication of backward citation *j* of patent *i* and $t_{i_{pub}}$ is the publication year of patent *i*. Note that Equation 10 is a linear combination of Equation 8 and Equation 6 and the expected correlation is now positive.

$$\frac{\sum_{i=1}^{SPC}\sum_{j=1}^{BC_{i}}t_{ji_{pub}}}{\sum_{i=1}^{SPC}\sum_{j=1}^{BC_{i}}1}$$ (10)

### Hypothesis 10

The *Average number of Forward Citations within 3 years of publication* is the numerator of the price index (Equation 7) and a good potential indicator of immediate importance and is used to test hypothesis 10. The metric is calculated using Equation 11 where SPC is the simple patent count, *FC*_*i*_ is the number of Forward citations for patent *i*, $t_{i_{pub}}$ is the publication year of patent *i*, $t_{ij_{pub}}$ is the publication date of forward citation *j* of patent *i*, and the function *IF(arg)* only counts the values if the argument is satisfied.

$$\sum_{i=1}^{SPC}\sum_{j=1}^{FC_{i}}IF(t_{ij_{pub}}-t_{i_{pub}}\leq 3)$$ (11)

The raw patent variables (dates, patent citations, NPL citations) for each of these metrics can be downloaded from www.patsnap.com in bulk for each patent set to allow for manipulation into the final forms shown in Equations 2–11. After each of the metrics are calculated for each domain, the k values (dependent variable) are plotted against the set of 28 data points for each patent metric (the dependent variables) for the 28 domains. A Pearson correlation coefficient and p value are also determined. A patent metric that correlates significantly in the expected direction with k is support for the related hypothesis and the concept that led to the hypothesis is thereby supported as well.

## Results

**Fig 1:**
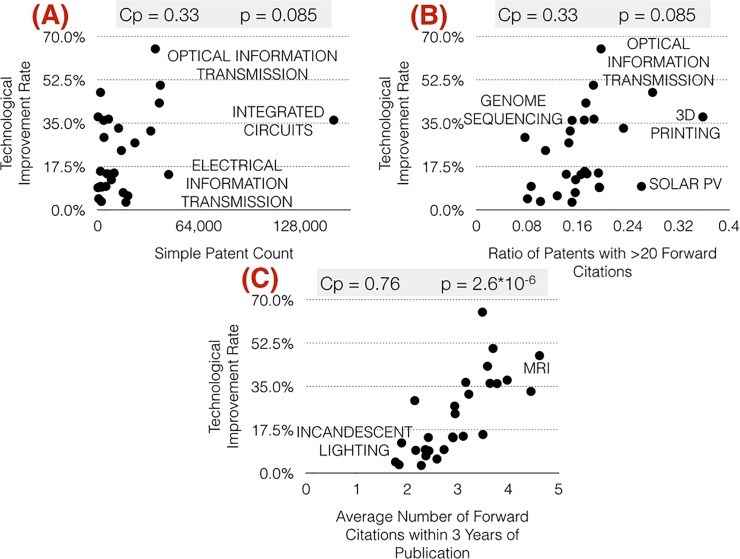
Technological Improvement Rates vs Simple Patent Count (A), ratio of patents with greater than 20 citations (B), and average number of forward citations within 3 years of publication (C); the Pearson correlation coefficient (c_p_), the null hypothesis acceptance (cutoff at p = 0.05) and the values of the independent variable for the domains having maximum and minimum values are shown in the upper right corner.

The relationship between a particular patent metric and the k values for all domains was examined graphically as well as statistically. Fig 1 shows examples of the three types of relationships between the k values and the patent metrics: *no relationship*, demonstrated in Fig 1(A) has a low correlation coefficient and high p-value, a *weak relationship* with a moderate correlation coefficient and p-value with an example in Fig 1(B), and a *strong relationship* with a high correlation coefficient and low p-value as in Fig 1(C).

Fig 1(A) shows a plot of the k values and simple patent count and exhibits no clear trend or relationship. The Pearson correlation coefficient between the two variables is 0.33, however the p value is a relatively high 0.085 so the correlation could easily be due to the random variation in the data. The combination of the statistical tests and the lack of a discernible trend in Fig 1(A) indicate that there is not a reliable relationship between the number of patents in a technological domain and the associated k. Thus, in this form effort in a domain surprisingly shows *no statistically significant relationship* with technological improvement in a domain.

Fig 1(B) is an example of a weak relationship between a patent metric, the % of patents with greater than 20 citations, and the k values. There seems to be a slight visual trend in the figure, the Pearson correlation is a moderate 0.39 and the p-value is slightly lower than is generally accepted for statistical significance, at 0.043. This indicates a *weak relationship* between the values for this patent metric and the k values for the 28 technological domains.

Contrastingly, Fig 1(C) shows the relationship between k and the average number of forward citations within 3 years of publication per patent in a domain. The Pearson correlation coefficient between the two variables is 0.76, and the p value is 2.6*10–^6^, indicating that the correlation is quite unlikely to be due to random scattering of the data. The combination of the statistical tests and the visible trend in Fig 1(C) indicate that there is a *strong relationship* between the average citations in the first three years to the patents in a technological domain and the associated k value.

All of patent metrics discussed in sections 2 and 3 were tested using this approach and the summary statistics and correlation coefficients are given in Table 2. The last two rows give the correlation between k and each specific patent metric (shown in the first column on the left and numbered across the top row). These results show k correlations with five of the patent metrics have p values < 0.01 indicating that total forward citations (column 2), average patent publication year (column 5), average age of backward citation (column 6) and especially mean publication date of backward citations (column 9) and average forward citations in the first three years (column 10) have strong correlations with k that are not at all likely due to noise in either the patent or rate data sets. We briefly note here the specific results and their relationship to the concepts and hypotheses from section 2 and interpret the results more fully in the discussion section.

**Table 2:** Summary Statistics and Correlation Matrix.

Variable	Mean	SD	Min	Max	(1)	(2)	(3)	(4)	(5)	(6)	(7)	(8)	(9)	(10)
(1) Simple Patent Count	18259	29110	154	149491	*1*.									
(2) Average number of forward citations	11.80	3.32	6.12	22.08	*0*.*01*	*1*.								
(3) Ratio of patents with cited by over 20	0.17	0.06	0.08	0.36	*-0*.*03*	*0*.*96*	*1*.							
(4) NPL Ratio	0.17	0.15	0.04	0.84	*-0*.*1*	*-0*.*25*	*-0*.*24*	*1*.						
(5) Average publication year	2000.7	2.9	1994.8	2006.7	*0*.*19*	*0*.*11*	*0*.*09*	*0*.*51*	*1*.					
(6) Average Age of backward Citation	10.70	3.44	6.66	18.33	*-0*.*18*	*-0*.*37*	*-0*.*22*	*-0*.*14*	*-0*.*23*	*1*.				
(7) Price Index (3 years)	0.26	0.05	0.18	0.35	*0*.*29*	*-0*.*37*	*-0*.*48*	*0*.*55*	*0*.*51*	*-0*.*52*	*1*.			
(8) Ratio of Backward Citations to Other Domains	0.10	0.04	0.02	0.20	*0*.*55*	*-0*.*03*	*-0*.*04*	*-0*.*39*	*-0*.*2*	*-0*.*28*	*0*.*13*	*1*.		
(9) Mean publication date of backward citations	1990.0	5.0	1981.1	1997.8	*0*.*23*	*0*.*31*	*0*.*21*	*0*.*4*	*0*.*74*	*-0*.*82*	*0*.*65*	*0*.*08*	*1*.	
(10) Average forward citations within 3 years	2.96	0.77	1.77	4.62	*0*.*26*	*0*.*77*	*0*.*64*	*-0*.*03*	*0*.*4*	*-0*.*73*	*0*.*27*	*0*.*13*	*0*.*74*	*1*.
K-Value correlation with Patent Metric	0.23	0.17	0.03	0.65	*0*.*33*	***0*.*48***	*0*.*38*	*0*.*2*	***0*.*54***	***-0*.*59***	*0*.*39*	*0*.*11*	***0*.*72***	***0*.*76***
*P-value*					*0*.*085*	***0*.*009***	*0*.*043*	*0*.*303*	***0*.*003***	***0*.*001***	*0*.*039*	*0*.*567*	***1*.*7E-05***	***2*.*6E-06***

Concept A, that effort is an important determinant of relative progress rates among domains surprisingly failed to achieve statistical empirical support. The hypothesis derived from this concept is tested in column 1 above and achieves a p value of. 095: this is above the normal cutoff for statistical significance. On the other hand, Concept B that technological improvement rates are higher in domains with more important/cited patents in a domain is supported. The hypotheses derived from this concept (H2 and H3) are both supported—see columns 2 and 3. The total forward citations (column 2) correlation is 0.48 and has a p value of. 009 which is relatively strong whereas the fraction of patents with more than 20 citations has a more modest correlation of 0.38 with p value of 0.043.

Concept C, which states that domains with closer connections to science improve more rapidly is surprisingly not supported statistically by the results. The test of hypothesis 4 is shown in column 4 of Table 2 and shows poor correlation (Cp = 0.2, p = 0.3). We were surprised enough by this result to test it again (see EC) with only the 100 most highly cited patents in the domains rather than our total set of patents (with less than 100% relevancy) but found even weaker correlation (Cp = -0.03, p = 0.86) for the clean Top100 patent sets. The essentially zero correlation between k and NPL for these clean and most important patents in a domain supports the earlier finding and will be discussed further below.

Concept D—Recency- and hypothesis five that is derived from it (domains with newer patent sets should improve more rapidly) does achieve firm empirical support. The test of this hypothesis is shown in column 5 above and demonstrates strong correlation of 0.54 with a p value of 0.003. Likewise, concept E—Technology improvement is enhanced by increased immediacy of use and knowledge base- is supported strongly. The hypotheses derived from it (H6 and H7) are tested in columns 6 and 7 in Table 2. Backward citation immediacy (column 6) shows strong expected (negative) correlation of -0.59 with a very strong p value (0.001) and forward citation immediacy (column 7) is supported but the correlation of 0.39 and p = 0.039 values are not as strong as for H6.

Concept F breadth of knowledge led to H8: domains that cite other domains more frequently will improve more rapidly. This hypothesis is tested in column 8 and does not show any sign of correlation with Cp = 0.11 and p = 0.57. The result of testing the combined recency and immediacy hypothesis is shown in column 9 to achieve a very strong correlation (Cp = 0.72, p = 1.7 x 10^−5^) with excellent explanatory power. Column 10 tests the hybrid of immediacy and importance and also shows a very strong correlation (Cp = 0.76, p = 2.6x 10^−6^) with perhaps even more explanatory power. The immediate importance metric has the strongest correlation of any of our patent metrics with the technological improvement rate.

Although seven correlations have p values less than our desired cutoff of 0.05, it is obvious that a number of them contain duplicated information and cannot be useful independently. A very clear example is seen for items 2 and 3 which both are designed as measures of importance and have a cross-correlation near 1 (Cp = 0.96). Not surprisingly, the combined/hybrid metrics have significant cross-correlations with other significant variables. The recent immediacy metric (column 9) shows cross-correlation greater than 0.6 with recency (column 5) as well as both immediacy metrics (6&7) as well as with the immediate importance metric (column 10). The immediate importance (10) metric has correlations greater than 0.6 with both importance metrics (columns 2&3) as well as the backward citation immediacy (column 6), and the recent immediacy metric (column 9), but not the forward citation immediacy metric (column 7). We will return to the issue of overall correlation with multiple regression models shortly but it is useful to first present results concerning robustness of the correlations.

### Robustness Testing

An important issue is whether our 28 domains contain significant selection bias. It is possible that domains we have not yet studied could change our results. Although this concern cannot be fully answered, one way to examine this issue is to look at correlations with smaller subsets of the 28 domains. We proceeded (see supporting material) with a relatively stringent test by randomly separating the set of 28 domains into 2 independent sets of 14 domains (with no domains repeated twice) and the correlation coefficients were re-calculated using only 14 domains each time. This trial was then completed 10 times for a total of 20 different sets of 14 domains and corresponding correlation coefficients. To examine each variable, the mean and standard deviation of the values were calculated, with the signal (r) to noise (sigma) values taken as a measure of robustness. Table 3 shows the summary of the domain selection robustness for all 10 metrics from Table 2.

**Table 3:** Summary of Domain Robustness Analysis.

Patent Metric	Correlation for all 28 domains	Standard Deviation of Correlation for 14 domains	Correlation / Standard Deviation (absolute value)
*(10) Average Cited by within 3 years*	*0*.*76*	*0*.*073*	*10*.*368*
*(9) Total mean publication date of backward citations*	*0*.*72*	*0*.*090*	*8*.*000*
(6) Average Age of Citation	-0.59	0.103	5.678
(5) Average publication year	0.54	0.128	4.178
(2) Average number of forward citations	0.48	0.136	3.567
(7) Price Index (3 years)	0.39	0.185	2.114
(3) Ratio of patents with cited by over 20	0.38	0.200	1.923
(1) Simple Patent Count	0.33	0.195	1.695
(4) NPL Ratio	0.2	0.152	1.326
(8) Ratio of Cites to Own Domains	0.11	0.257	0.440

Not surprisingly, the correlations with the lowest p values were the most robust to this domain selection test. Given the severity of the test in removing ½ of the domains, there is quite good consistency of the correlations of the metrics on the rate of improvement for each of the metrics with p values < 0.01. In particular, the immediate importance metric of average forward citations within 3 years of publication is remarkably consistent across 20 different correlation tests, indicating that the strength of that signal is not likely to be due to the selection of these specific 28 domains. In the linear regression analysis below, we only use the 5 metrics that are shown to be strongest by this test and by their p values for the entire 28-domain correlation.

### Regression Analysis

The five metrics identified above as showing statistically significant and robust correlation with the k values were included in linear regression models for predicting the technological improvement rate. Numerous regression models were tested using a combination of these variables and the most informative are shown in Table 4.

**Table 4:** Least Squares Linear Regression Models for Predicting Technological Improvement Rates with R^2^ shown for each model and the coefficients shown for each metric included in the model and its p value.

Variable/Models	A	B	C	D	E	F	G	H
(2) Average number of forward citations					-0.01		0.014	0.015
*p-value*					*0*.*34*		*0*.*044*	*0*.*043*
(5) Average publication year				0.02				0.024
*p-value*				*0*.*05*				*0*.*005*
(6) Average Age of Citation			-0.003			0.0004		-0.018
*p-value*			*0*.*704*			*0*.*969*		*0*.*013*
(9) Total mean publication date of backward citations		0.01				0.024	0.020	
*p-value*		*0*.*12*				*0*.*0067*	*9E-5*	
(10) Average Cited by within 3 years	0.16	0.11	0.15	0.14	0.19			
*p-value*	*1E-5*	*0*.*02*	*0*.*009*	*4E-5*	*0*.*0003*			
Intercept	-0.23	-20.44	-0.19	-31.12	-0.21	-47.66	-41.37	-47.1
*p-value*	*0*.*02*	*0*.*12*	*0*.*37*	*0*.*05*	*0*.*03*	*0*.*01*	*9E-5*	*0*.*005*
**Total R2**	**0.53**	**0.57**	**0.58**	**0.64**	**0.55**	**0.51**	**0.59**	**0.59**

Model A in Table 4 is for the single variable of Forward Citations within 3 years of publication and has a R^2^ of 0.53 which indicates that this single variable can “explain” more than ½ of the variation in k across the domains. It is the most powerful of the variables tested and we use it as the basis for Models B through F in Table 4. Model B combines the two variables (10 and 9) that are individually the most strongly correlated with the k values in the domains. While some improvement in R^2^ (0.57) is seen relative to model A, the p values for the coefficient of variable 9 and the intercept indicate that the improvement could well be due to over-fitting. Model C adds the strongest immediacy metric (#6) to the immediate important metric (#10) and similarly improves R^2^ but with p values that make over-fitting a significant concern. Note that the only p values that are strong in both models B and C are for the coefficient for the immediate important metric indicating again the strength of this variable.

Model D combines immediate importance with recency (patent publication date- metric # 5). Despite this variable having the fourth highest correlation with the k-values, it is the first to add significantly to R^2^ (0.64) and does so with p values that make over-fitting unlikely. The combination of the strongest importance metric (#2) with the immediate importance metric is model E and this (like models B and C) gives very modest improvement in R^2^ with p values that raise significant concern about over-fitting. Models F and G leave out the strongest metric (immediate importance) and start with the second strongest (recent immediacy, #9) as the basis. Model F combines the recent immediacy metric and the strongest immediacy metric (average age of backward citation, #6): the p value for the coefficient on metric #6 indicates over-fitting for this variable is very likely. Model G, on the other hand, incorporates the strongest importance variable (forward citations, #2) with the recent immediacy metric (#9) and achieves the (tied for) second best R^2^ along with p values that make over-fitting unlikely. Model H uses neither of the two strongest (hybrid) metrics but instead each of the strongest singular metrics for the three concepts and also achieves the (tied for) second best R^2^ (0.59). Perhaps most interesting is that the p values for *all three* coefficients in Model H indicate significance.

Overall, the results in Table 4 indicate, not surprisingly, that the best multiple regressions were those using variables that are not highly cross-correlated. Examination of Table 4 shows that of the multiple variable models above only Models D, G and H (which are the only models without over-fitting indications) use variables with cross-correlation < 0.4 (whereas the other multiple variable models- B, C, E, and F- employ variables with cross-correlations >0.6). The overall results (and the cross-correlations) also show that the three models with the best fits (D, G and H) each combine importance, recency and immediacy even though they employ different metrics. These results are evidence that *all three concepts* have a role in explaining variation in k among a variety of technological domains.

An important issue is the ability of the correlations to work in the future not just in the past. A second robustness test examines the predictive capability of the correlations by testing how sensitive the patent metrics correlations were to variations in time. In order to do this, the patent metrics were analyzed for only patents from a variety of time frames that were less than the total time frame. The time frames were analyzed to see how far back from 2013 they could be analyzed and still find similar correlations as the patent metrics show during the entire time frame (1976–2013) and are shown in the supporting information. Ultimately the two strongest and most robust patent metrics are robust to time up to 12 years prior to the experiment reported in detail here, indicating a promising amount of predictive capability.

## Discussion

### Interpretation of results

The major finding of the present study is robust, strong correlations between technological improvement rate and patent metrics for a wide variety of technological domains. An unacceptable interpretation is that the metrics that are strongly correlated with technological improvement rate *cause* the faster rate of improvement. However, it is reasonable to postulate (as we did in the hypotheses development) that the concepts being tested by the metric (for example importance, recency and immediacy) are causing both the increase in the metric and an increase in the rate of progress.

As discussed in the literature review supporting hypothesis development, the use of forward citations for estimating importance of a single patent has been well established. The results reported here show that the *average forward citation rate to patents in a domain is strongly correlated with the differing rates of progress in these domains*. This represents significant additional support for the usage of patent citations to assess patent importance. Moreover, interpreting that variations in both *forward citation frequency and technological progress in a domain are due to the importance of the patents in the domain* receives support from these results.

Average publication date correlating strongly with technological improvement rate in the variety of domains is also not surprising. Although technology overall being hyper-exponential and thus many rates might increase over time [69] can be part of the explanation, a Darwinian interpretation is probably also important. If there are a large number of potential domains being developed at all times, it is likely that only the domains that improve more rapidly than the current state of the art will be developed further, and thus patented, diffused and studied by technological change researchers. Thus, the recency of emergence of a technology should correlate with higher rates of improvement and such domains will automatically have a later average patent publication date accounting for the robust correlation between these parameters that was found.

The concept of immediacy, first developed by Price [57] as a key characteristic that distinguished rapidly developing scientific fields from fields that were not developing as rapidly, was extended here to suggest an analogous effect in technology. This concept is not the same as recency since immediacy refers to the pace of knowledge use (backward and forward) at all points in time not just presently. Nonetheless, more immediate use of patents in other domains means that the knowledge base (at all times) is more current than for a less immediate domain so some of the causal benefits of recency described in the previous paragraph apply. Despite the interaction of the recency and immediacy concepts, the results indicate that they *independently* drive faster technological improvement. More rapid knowledge incorporation as signaled by the immediacy metrics does appear to lead to higher technological improvement rates across domains. The fact that all three concepts (importance, recency and immediacy) have independent effects on the technological improvement rates is supported by the multiple regression results in Table 4 and the cross-correlation results in Table 2.

One of the most important implications of our findings is that patents do contain much information relevant to distinguishing among technological improvement rates in the 28 domains investigated here. Hypothesis 0 is strongly confirmed by the high R^2^ values for the regressions and the multiple strong correlations with patent variables: these findings clearly demonstrate that patents do contain information that is essential to increases in technological improvement rate.

This result is much more aligned with the position that patents are the major data source for technological progress than the contrarian position that patents have very little to do with technological progress. Moreover, analysis of why the explanatory power is not even higher (the R^2^ indicates that more than 1/3 of the variation in k is not explained by combinations of the best variables we have examined) indicates that perhaps only a small part of the issue is lack of information in patents. A Monte Carlo analysis was performed (see supporting information) for the correlations based upon estimating the k value standard deviation for each domain. Although the standard deviation estimates are subjective, the results suggest that R^2^ even with a perfect theory would be reduced to 0.8 to 0.84 due to the imperfect ability to measure k. This indicates that estimating k introduces sufficient noise to account for about ½ of the imperfection found with our model fit to the data. The imperfections in our patent sets representing the domains (62) can diminish the correlations and the possibilities of inconsistent patent writing practices among domains, of better but unknown metrics, for non-linear relationships contributing to imperfect linear correlations and for real effects from textual facts contained in the patents all appear also likely to diminish correlation. Therefore, improvement contributions not captured in patents is definitely less than the contribution of k estimation noise and may not be a significant factor in understanding the imperfections in the regressions.

The results did not support three of the concepts for which we developed hypotheses about their potential influence on the relative rate of performance improvement: effort within a domain, the breadth of knowledge used by a domain and the directness of the science link to a domain are the three unsupported concepts that will each be discussed now. The reasons for the failure to find correlation in each of these cases can be of two kinds: 1) that the concept in fact does not drive *differences in technological progress among domains* and 2) that the metric(s) we have tested do not appropriately represent the concept.

It is a truism that human effort is needed to get any technological progress. However, relatively higher effort *within* a domain does not necessarily lead to relatively greater progress in that domain since so much work has shown the importance of “spillovers” from other domains and from science that are *not* dependent upon effort within the domain. Indeed, knowledge flows from citations indicate that all domains are more dependent upon developments in other domains (spillover) and scientific findings not arising from the effort within a domain than they are to effort within the domain [68]. Thus, the first type of reason (non-viable concept) above is quite possible for the effort in a domain concept. The second reason is also potentially operative for the effort concept at least because effort variables are prolific (revenue, R&D spending, production experience and man-hours have been suggested).

Although breadth of utilized knowledge is a reasonable concept to hypothesize as driving differences in performance improvement among domains, the failure of our test (no sign of correlation) is not as surprising as for the other two failed concepts. This is because a number of tests of breadth of knowledge (on importance of—citations to- individual patents) using various metrics (including number of patent classes per patent) have shown weak and sometimes contrary results [61,63,70,71]. Moreover, in the present work other metrics were tested (number of patent classes for citations, etc., see supporting information) and *none* of them showed significant correlation. It appears that broad utilization of knowledge is a primary and important feature of technological development but that *knowledge breadth differences do not drive differences in performance improvement dynamics* among domains {and perhaps not among important and unimportant patents. Spillover seems to be generally important in individual patents and even then it appears that an intermediate amount of breadth of knowledge may be optimal [72].

To question whether science has any impact on technological progress is not a reasonable line of inquiry but the process by which science impacts technology is not yet fully established. Thus, it is not clear that the impact of science should have different impact on performance improvement among domains nor that the impact of science is measured well by citations in patents to scientific articles. Price argued quite early [42] that scientific impacts would largely come through education of inventors and that the more direct impact was in the reverse direction-of technology on scientific empirical tools. He argued for very long lags for the impact of science on technology and this might reasonably imply that our finding of no short-term effects is expected. A more recent concept for the impact of science on technology [49] is that science acts as a map that makes technological search by inventors more effective. Fleming and Sorenson [49] also developed the concept to show that science would then be more useful in problems where interactions of components is more complex (more component interactions). If we extrapolate this concept to understand differences in domains, it is appealing to think that science is more useful in more complex domains; however, qualitative [9] and quantitative [73] concepts have suggested the rate of advance should be slower in more complex domains. This reasoning leads to a possible *negative* correlation of scientific references with progress rate and this could negate any positive effects and thus this framework for understanding the interaction of science and technology is also potentially consistent with our findings of no effects.

Some authors suggest that more heavily cited patents themselves cite more scientific articles [53]. More detailed study of specific cases of science and technology [50,51,74] has found the science/technology exchange mechanism to be deep and involve personal communication and other forms of social capital. In Murray’s cases [50,51], there were scientific papers and patents written by the same individuals but there was no indication in the patent citations that captured the intense interaction. Thus, the metric we use may not capture the effect of science on technology by domain (if one even exists).

Overall, it appears that the concept-that breadth of knowledge affects differential improvement rates among domains- is not viable with any metric. On the other hand, we feel that the evidence suggests that the concept- differential science links explain some of the performance rate differential- remains quite viable as a potential explanation despite the failure of our framework to find the effect. The most we can conclude about the third concept- differential effort among domains explain some of the performance rate differential- is that our failure to find such an effect could be due to non-viability of the concept or to metric/framework shortfalls.

#### Implications to research on and theories of technical change

One clear implication of the work reported here is that the patent data contains information that can be used to understand the relative rate of improvement among technological domains. The results also strongly support the current practice of using forward citation counts to represent the importance of patents while giving the first indication that importance assessed this way can be extended to entire domains by simple averages across the domains. The work reported here also suggests that little used metrics such as the average patent publication date and the average age of backward citations are quite useful in studying differences among domains. We also introduced two new fairly simple-to- calculate metrics, the average number of forward citations to a group of patents in the first three years after patent publication and the average publication date of the backward citations from a group of patents, that were shown to be particularly powerful in distinguishing among groups of patents. We believe these metrics should be useful to others interested in understanding differences between groups of patents beyond our focus here on understanding the relative rate of progress among a well-defined set of technological domains.

The individual significance of importance, recency and immediacy on the relative rate of progress in technological domains is conceptually significant. Although we did not create any of these concepts, we believe we have distinguished more carefully among them: the empirical work establishes the distinction among these three concepts as meaningful. We suggest that each of these concepts can have causal implications in other technical change phenomena and might fruitfully be more widely studied in other contexts.

The strong explanatory power of models that combine all three concepts also has conceptual implications. A possible connection to prior concepts is with the conceptual frameworks that attribute much of technological change to discontinuities; however, we believe it is important to make the connection with some care. Although not always clearly specified, these concepts often seem to focus on a sharp technological discontinuity whereas our results show that dynamic domains remain such. For our 28 domains, many of the more rapidly improving cases have shown such behavior for more than the 35+ years for which we were able to obtain the corresponding patents and none of these have appreciably yet slowed in performance improvement. A second reason for care is that many of the prior examples of qualitatively selected very important inventions are represented by a large set of patents in this paper- perhaps even a domain such as integrated circuits with its almost 150,000 patents.

The preceding points suggest that a potentially better way to make the connection between technological discontinuities and domains with patents of high importance, recency and immediacy is to assert that the discontinuity of interest is the *emergence* of new dynamic domains; however, even this discontinuity focus may obscure the fact that dynamic domains (such as integrated circuits or wireless transmission among our domains) do not have their major economic and societal impact at emergence. Their disruptive and apparently discontinuous impacts instead often occur after decades of dynamic improvement. As such a domain continues to rapidly improve, the performance of artifacts in the domain rapidly rises so that more and more application fields are affected in the manner of general purpose technologies [75]. Although the changes in given fields are quite disruptive, the technological performance has grown over many years. Rapidly improving technological domains can in very few years go from being non-competitive in an application field to dominant: this makes such technological domains important in observed discontinuities. Thus, the implication to theories about discontinuities from the current work is to consider domains that have rapid rates of improvement as major sources of discontinuous change. This work has demonstrated that such technological domains have relatively higher levels of important, recent and immediate patents.

One more speculative conceptual contribution is largely based upon the failed correlations as well as the successful ones: we call this concept the rising sea metaphor. Our results show that measurements at the *domain level* of importance, recency and immediacy correlate strongly with the rate of progress in *that domain*; however, the results also indicate that *effort and science* links measured at the *domain level* do not correlate with the rate of progress in the domain. The rising sea conceptualization imagines the contributions of science and inventions from all domains to be *equally* available to all domains but the ability of domains to convert that rising sea to performance improvement is strongly dependent upon *fundamental characteristics of that domain*. Such fundamental characteristics could involve the intensity of interactions among components in the domain [9,73] as well as the impact of feature scale on performance of artifacts in the domain [76,77].

#### Implications for technology strategy for firms

The technological improvement rate of a domain can be very useful in understanding the potential of a specific technology particularly if one compares it to the improvement rate of competitive and complementary technological domains. This is because the improvement rates are reasonably consistent across time [5] so a domain that is improving much more rapidly than a competitive domain will almost always eventually (even shortly) dominate the competitive markets (except for a few resistant niches). Thus, quantitative technology improvement rates are helpful in understanding the future of technology from the component level to entire industries. While having reliable quantitative rates of improvement can be powerful, determining the improvement rate of even one domain can be very difficult, time consuming, and is often not possible depending on the availability of data. These issues are the main reason why reasonably reliable improvement rates have been found for only a small percentage of possible domains.

The results of the research reported here are correlations robust to the domains analyzed and consistent for 12 years into the future (2001–2013). These findings statistically (in a robust way) reflect what is likely to happen—or at least what is happening now- in performance trends. The process of estimating a technological improvement rate given a domain of interest works as follows:

1. Select a domain of interest
2. Use the COM [66,68] to select a set of patents that represent the domain
3. Calculate the average number of forward citations in 3 years (column 10 in Table 2) and the average publication year (column 5) of the patent set
4. Use the predictive model D in Table 4 to estimate the improvement rate

The R^2^ of this predictive model is 0.64, so 64% of the variation in the improvement rate can be explained by the variation in the patent metrics included in the model. This type of estimate can be made in less than 3 hours (at least by an experienced COM user) and is probably nearly as accurate as an estimate that might take more than 30 hours of data search (and might not be possible to find in infinite time). A major implication from the research reported here is the potential to greatly expand the usage of technological improvement rates in technology strategy and research policy. Some useful approaches include:

- Quantitatively monitoring improvements at all phases of technological maturity to understand if large (unexpected) changes have occurred.
- Monitoring improvement rates in key competing (threat and opportunity) technologies.
- The patent based approach to estimation of improvement rates described above can be the basic approach to the monitoring task and it might be applied even very early in the technology’s history possibly even before the start of commercial production as long as sufficient patenting has started.
- Often times a competing technology has been used in other application fields and thus improvement rates might be found from actual data but using the patent based approach above would still be useful to improve the robustness of the estimate.

Based upon the prior discussion, relative rates of technical performance increase can have large implications for the future viability of component technologies in products and systems as well as the viability of industries and thus have great importance to forward-looking firms. Acquisition strategy, product component technology choice and appropriate research goals could be informed by improved understanding of the probable improvement potential of relevant technologies. Moreover, the results of performance improvement monitoring have implications for choosing technologies that should receive research funding from firms and governments and for choosing ventures in which to invest risk capital.

## Conclusion

This paper represents the first statistically significant comparison between metrics that were derived from individual patent sets from a group of technological domains and the performance improvement rates of the same individual domains. This was done to test hypotheses derived from existing theories of technological change, to initiate predictive theory development and to establish a stronger practical basis for technology strategy and planning for firms and governments. The strong correlations (r = 0.76 for the strongest case) and multiple regressions (R^2^ = 0.64) establish an important empirical finding: patents do contain much significant information relevant to quantitatively determining the differences in technological improvement rates.

The main theoretical implications of the findings reported here are that average importance, recency and immediacy of the patents in a domain each individually drive higher improvement rates and that these concepts are independent enough that models that combine all three are robust predictors of a domains improvement rate. The prediction models apparently provide good evidence of what change is currently happening and meaningful forecasts of the future within the specified robust time frame of 12 years, however past results are not always indicative of future returns and the estimations of the k’s are subject to the same disclaimer. Thus, the potential weaknesses (and possibly unrecognized at present strengths) of the practical application of the results of this research will only be known if and when widespread application occurs.

## Supporting Information

S1 File Table A. Patents obtained, relevancy and k-value for each of the 28 domains. **Table B.** Raw values of 10 variables for 28 domains. **Table C.** Raw values of extra variables for 28 domains. **Table D.** Correlation values for all variables for 28 domains. **Table E.** Domain Robustness Tests for 10 variables for 20 trials (14 domains each). **Table F.** Time Robustness Analysis for 2 Patent metrics showing Pearson correlation and p-value.
(DOCX)
