# Correction: Quantitative Determination of Technological Improvement from Patent Data

**DOI:** 10.1371/journal.pone.0151931

**Published:** 2016-03-21

**Authors:** Christopher L. Benson, Christopher L. Magee

Fig 1 appears incorrectly in the published article. Please see the correct Fig 1 and its legend here.

**Fig 1:**
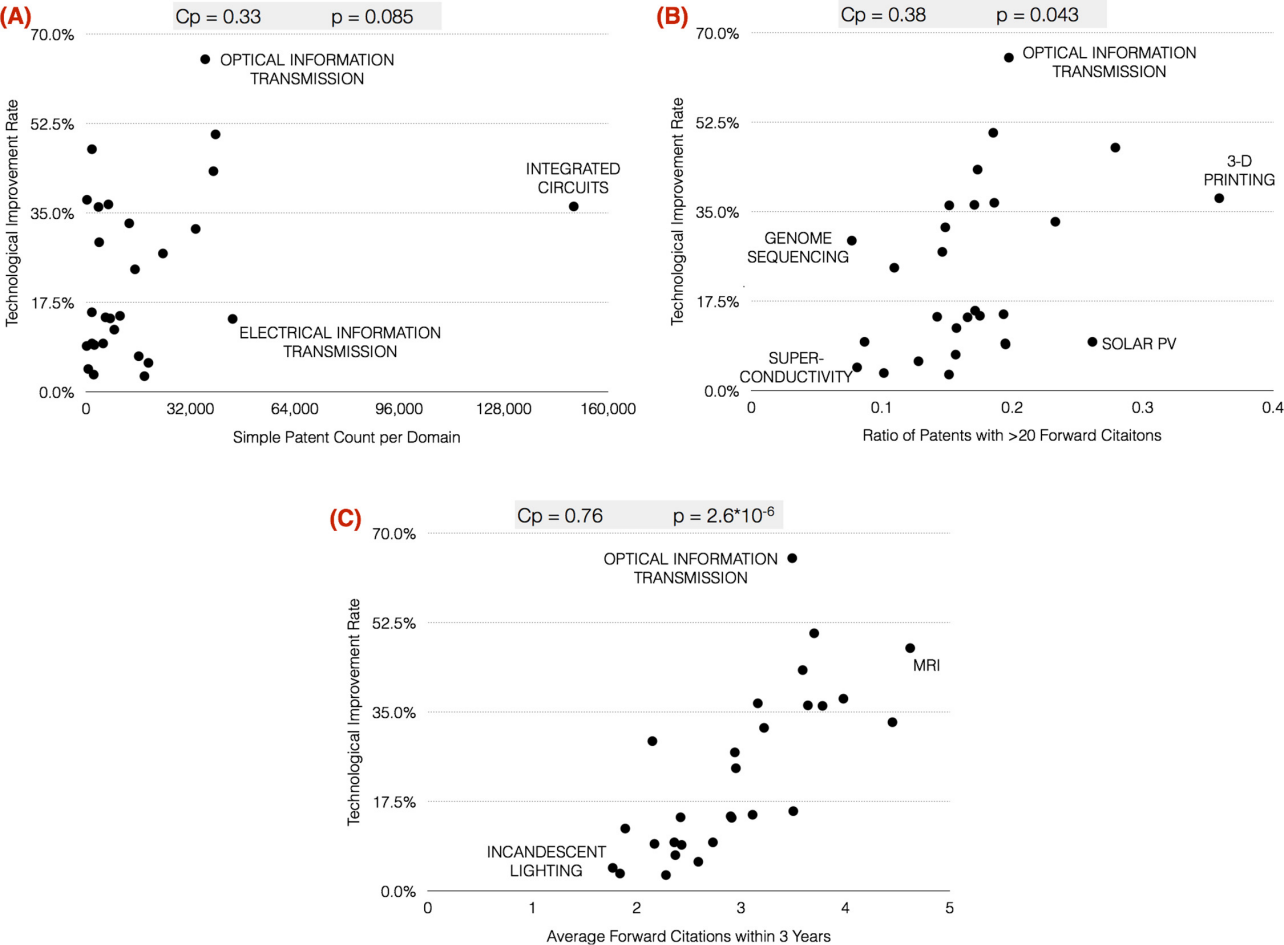
Technological Improvement Rates vs Simple Patent Count (A), ratio of patents with greater than 20 citations (B), and average number of forward citations within 3 years of publication (C); the Pearson correlation coefficient (cp), the null hypothesis acceptance (cutoff at p = 0.05) and the values of the independent variable for the domains having maximum and minimum values are shown in the upper right corner.

There is an error in the second sentence of the third paragraph in the Results section. The correct sentence is: There seems to be a slight visual trend in the figure, the Pearson correlation is a moderate 0.38 and the p-value is slightly lower than is generally accepted for statistical significance, at 0.043.

Table 4 appears incorrectly in the published article. Please see the correct Table 4 and its legend here.

**Table 4:** Least Squares Linear Regression Models for Predicting Technological Improvement Rates with R2 shown for each model and the coefficients shown for each metric included in the model and its p value.

Variable/Models	A	B	C	D	E	F	G	H
(2) Average number of forward citations					-0.01		0.014	0.015
*p-value*					*0*.*34*		*0*.*044*	*0*.*043*
(5) Average publication year				0.0155				0.024
*p-value*				*0*.*05*				*0*.*005*
(6) Average Age of Citation			-0.003			0.0004		-0.018
*p-value*			*0*.*704*			*0*.*969*		*0*.*013*
(9) Total mean publication date of backward citations		0.01				0.024	0.020	
*p-value*		*0*.*12*				*0*.*0067*	*9E-5*	
(10) Average Cited by within 3 years	0.16	0.11	0.15	0.141	0.19			
*p-value*	*1E-5*	*0*.*02*	*0*.*009*	*4E-5*	*0*.*0003*			
Intercept	-0.23	-20.44	-0.19	-31.197	-0.21	-47.66	-41.37	-47.1
*p-value*	*0*.*02*	*0*.*12*	*0*.*37*	*0*.*05*	*0*.*03*	*0*.*01*	*9E-5*	*0*.*005*
**Total R2**	**0.53**	**0.57**	**0.58**	**0.64**	**0.55**	**0.51**	**0.59**	**0.59**
