# Supplementary material for: Quantitative Determination of Technological Improvement from Patent Data
Source: PLoS One. 2015 Apr 15;10(4):e0121635. doi: 10.1371/journal.pone.0121635 (PMC4398537; doi:10.1371/journal.pone.0121635)
Supplement: S1 File — Table B. Raw values of 10 variables for 28 domains. Table C. Raw values of extra variables for 28 domains. Table D. Correlation values for all variables for 28 domains. Table E. Domain Robustness Tests for 10 variables for 20 trials (14 domains each). Table F. Time Robustness Analysis for 2 Patent metrics showing Pearson correlation and p-value. (DOCX) [file pone.0121635.s001.docx]

**Supporting Information for:**

**Quantitative Determination Of Technological Improvement From Patent Data**

In order to supplement the information contained in the paper, some aspects of the data and additional analyses are reported here.

A summary of the key data about the 28 technological domains we examined is shown in Table A. The k value determination is covered in Magee et al (2014) and the patent class size and relevancy are from Benson and Magee (2014b)

**Table A Patents obtained, relevancy and k-value for each of the 28 domains**

| **Technological domain** | **k** | **Size of patent class** | **Relevancy** |
| --- | --- | --- | --- |
| 3D-Printing (industrial stereolithography) | 37.60% | 251 | 93% |
| Aircraft Transport | 12.20% | 8629 | 79% |
| Camera Sensitivity | 15.60% | 1744 | 86% |
| Capacitor Energy Storage | 14.60% | 5944 | 84% |
| Combustion Engines | 5.70% | 19094 | 96% |
| Computed Tomography (CT) | 36.70% | 6817 | 88% |
| Electric Motors | 3.10% | 17869 | 86% |
| Electrical Energy Transmission | 14.90% | 10375 | 86% |
| Electrical Information Transmission | 14.30% | 44910 | 67% |
| Electrochemical Battery Energy Storage | 7.00% | 16122 | 83% |
| Electronic Computation | 33.00% | 13204 | 97% |
| Flywheel Energy Storage | 9.00% | 154 | 70% |
| Fuel Cell Energy Production | 14.40% | 7368 | 97% |
| Genome Sequencing | 29.30% | 3990 | 74% |
| Incandescent Artificial Illumination | 4.50% | 642 | 89% |
| Integrated Circuit Information Storage | 43.20% | 49018 | 81% |
| Integrated Circuit Processors | 36.30% | 149491 | 81% |
| LED Artificial Illumination | 36.20% | 3792 | 85% |
| Magnet Resonance Imaging (MRI) | 47.50% | 1778 | 86% |
| Magnetic Information Storage | 31.90% | 33576 | 93% |
| Milling Machines | 3.40% | 2315 | 93% |
| Optical Information Storage | 27.10% | 23543 | 82% |
| Optical Information Transmission | 65.10% | 36494 | 82% |
| Photolithography | 24.00% | 14975 | 87% |
| Solar Photovoltaic Energy Generation | 9.50% | 5203 | 85% |
| Superconductivity | 9.50% | 1776 | 85% |
| Wind Turbine Energy Generation | 9.20% | 2498 | 94% |
| Wireless Information Transmission | 50.40% | 39675 | 94% |

The raw values of each of the tested variables for each of the 28 domains are shown in Table B – these can be used for corroborating (or not) the research results reported and for further analysis.

**Table B Raw values of 10 variables for 28 domains**

| **FormalDependent Variable** | **(1) Simple Patent Count** | **(2) Average number of forward citations** | **(3) Ratio of patents with cited by over 20** | **(4) NPL Ratio** | **(5) Average publication year** | **(6) Average Age of backward Citation** | **(7) Price Index (3 years)** | **(8) Ratio of Cites to Own Domains** | **(9) Total mean publication date of backward citations** | **(10) Average Cited by within 3 years** |
| --- | --- | --- | --- | --- | --- | --- | --- | --- | --- | --- |
| 3D-Printing (industrial stereolithography) | 251 | 22.08 | 0.36 | 0.14 | 2001.44 | 9.69 | 0.18 | 0.06 | 1991.75 | 3.98 |
| Aircraft Transport | 8629 | 10.25 | 0.16 | 0.10 | 1999.18 | 17.18 | 0.18 | 0.09 | 1982.00 | 1.89 |
| Camera Sensitivity | 1744 | 12.84 | 0.17 | 0.11 | 2000.48 | 6.66 | 0.27 | 0.06 | 1993.82 | 3.50 |
| Capacitor Energy Storage | 5944 | 12.13 | 0.18 | 0.11 | 2000.06 | 10.17 | 0.24 | 0.09 | 1989.89 | 2.90 |
| Combustion Engines | 19094 | 9.43 | 0.13 | 0.07 | 1998.41 | 13.88 | 0.27 | 0.12 | 1984.53 | 2.59 |
| Computed Tomography (CT) | 6817 | 13.35 | 0.19 | 0.16 | 2001.52 | 8.57 | 0.24 | 0.11 | 1992.96 | 3.16 |
| Electric Motors | 17869 | 10.19 | 0.15 | 0.07 | 1999.48 | 18.33 | 0.22 | 0.11 | 1981.15 | 2.28 |
| Electrical Energy Transmission | 10375 | 12.77 | 0.19 | 0.10 | 1999.56 | 8.68 | 0.24 | 0.14 | 1990.88 | 3.11 |
| Electrical Information Transmission | 44910 | 11.57 | 0.17 | 0.04 | 2000.11 | 11.08 | 0.25 | 0.20 | 1989.03 | 2.91 |
| Electrochemical Battery Energy Storage | 16122 | 10.14 | 0.16 | 0.18 | 1999.89 | 10.46 | 0.23 | 0.10 | 1989.43 | 2.37 |
| Electronic Computation | 13204 | 15.91 | 0.23 | 0.21 | 2002.09 | 6.75 | 0.28 | 0.10 | 1995.34 | 4.45 |
| Flywheel Energy Storage | 154 | 11.59 | 0.19 | 0.08 | 1995.97 | 14.90 | 0.21 | 0.04 | 1981.07 | 2.43 |
| Fuel Cell Energy Production | 7368 | 9.23 | 0.14 | 0.20 | 2005.19 | 12.00 | 0.26 | 0.07 | 1993.19 | 2.42 |
| Genome Sequencing | 3990 | 6.12 | 0.08 | 0.84 | 2006.69 | 12.94 | 0.35 | 0.02 | 1993.75 | 2.15 |
| Incandescent Artificial Illumination | 642 | 8.08 | 0.08 | 0.06 | 1994.83 | 13.36 | 0.22 | 0.06 | 1981.47 | 1.77 |
| Integrated Circuit Information Storage | 39018 | 12.56 | 0.17 | 0.14 | 2002.15 | 7.06 | 0.29 | 0.17 | 1995.09 | 3.59 |
| Integrated Circuit Processors | 149491 | 12.33 | 0.17 | 0.18 | 2002.98 | 9.51 | 0.30 | 0.15 | 1993.47 | 3.64 |
| LED Artificial Illumination | 3792 | 11.74 | 0.15 | 0.29 | 2006.57 | 8.74 | 0.32 | 0.03 | 1997.84 | 3.78 |
| Magnet Resonance Imaging (MRI) | 1778 | 18.94 | 0.28 | 0.20 | 2001.05 | 7.73 | 0.24 | 0.06 | 1993.32 | 4.62 |
| Magnetic Information Storage | 33576 | 10.80 | 0.15 | 0.08 | 1998.48 | 6.98 | 0.30 | 0.19 | 1991.50 | 3.22 |
| Milling Machines | 2315 | 8.69 | 0.10 | 0.06 | 1996.58 | 15.23 | 0.21 | 0.07 | 1981.35 | 1.84 |
| Optical Information Storage | 23543 | 10.14 | 0.15 | 0.09 | 2000.26 | 7.51 | 0.29 | 0.13 | 1992.75 | 2.94 |
| Optical Information Transmission | 36494 | 12.74 | 0.20 | 0.19 | 2001.26 | 8.86 | 0.27 | 0.13 | 1992.41 | 3.49 |
| Photolithography | 14975 | 9.08 | 0.11 | 0.22 | 2002.90 | 9.36 | 0.32 | 0.08 | 1993.54 | 2.95 |
| Solar Photovoltaic Energy Generation | 5203 | 14.99 | 0.26 | 0.23 | 1998.63 | 10.60 | 0.18 | 0.08 | 1988.03 | 2.73 |
| Superconductivity | 1776 | 7.77 | 0.09 | 0.44 | 1997.62 | 7.09 | 0.30 | 0.11 | 1990.53 | 2.36 |
| Wind Turbine Energy Generation | 2498 | 11.25 | 0.19 | 0.10 | 2002.83 | 17.28 | 0.19 | 0.06 | 1985.55 | 2.17 |
| Wireless Information Transmission | 39675 | 13.58 | 0.19 | 0.16 | 2004.39 | 8.97 | 0.27 | 0.07 | 1995.42 | 3.70 |
| Mean | 18258.82143 | 11.80 | 0.17 | 0.17 | 2000.74 | 10.70 | 0.26 | 0.10 | 1990.04 | 2.96 |
| SD | 29109.6619 | 3.32 | 0.06 | 0.15 | 2.91 | 3.44 | 0.05 | 0.04 | 4.99 | 0.77 |
| Min | 154 | 6.12 | 0.08 | 0.04 | 1994.83 | 6.66 | 0.18 | 0.02 | 1981.07 | 1.77 |
| Max | 149491 | 22.08 | 0.36 | 0.84 | 2006.69 | 18.33 | 0.35 | 0.20 | 1997.84 | 4.62 |

We also tested 13 other potentially important variables that could be of use in exploring more complex relationships than those we reported: these are shown below in Table C. In particular note the same variables applied to just the top 100 most cited patents in each field, these numbers allow a comparison between the total patents in a field and just the most important.

**Table C Raw values of extra variables for 28 domains**

| **FormalDependent Variable** | **Average Cited by within 5 years** | **Average number of UPCs cited (per patent)** | **Average number of IPCs cited (per patent)** | **Average number of backward citations** | **Top100 NPL %** | **Top100 Average number of forward citations** | **Top100 Average publication year** | **Top100 Average Cited by within 3 years** | **Top100 Average Cited by within 5 years** | **Top100 Average number of IPC classifications** | **Top100 Average number of UPC classifications** | **Average Age of Forward Citation** | **NPL >0**  **Ratio** |
| --- | --- | --- | --- | --- | --- | --- | --- | --- | --- | --- | --- | --- | --- |
| 3D-Printing (industrial stereolithography) | 6.90 | 24.86 | 82.00 | 20.42 | 0.06 | 45.24 | 1996.8 | 7.16 | 12.93 | 2.78 | 9.32 | 5.08 | 0.55 |
| Aircraft Transport | 3.15 | 12.97 | 24.59 | 15.46 | 0.01 | 77.78 | 1990.6 | 7.38 | 14.18 | 1.92 | 4.29 | 5.71 | 0.31 |
| Camera Sensitivity | 5.55 | 13.36 | 32.72 | 11.47 | 0.04 | 74.94 | 1995.4 | 17.71 | 29.51 | 2.98 | 6.19 | 4.46 | 0.39 |
| Capacitor Energy Storage | 4.75 | 9.77 | 20.34 | 13.07 | 0.03 | 106.61 | 1995.0 | 19.24 | 35.93 | 2.38 | 5.51 | 5.06 | 0.33 |
| Combustion Engines | 3.97 | 4.28 | 6.50 | 12.70 | 0.03 | 89.95 | 1992.7 | 19.46 | 33.68 | 3.55 | 3.86 | 6.21 | 0.18 |
| Computed Tomography (CT) | 5.12 | 9.86 | 18.66 | 12.72 | 0.03 | 140.75 | 1993.9 | 18.56 | 34.98 | 1.89 | 4.23 | 4.72 | 0.43 |
| Electric Motors | 3.69 | 2.22 | 3.86 | 14.49 | 0.02 | 85.74 | 1990.3 | 11.44 | 20.35 | 2.18 | 4.52 | 6.73 | 0.24 |
| Electrical Energy Transmission | 4.98 | 3.04 | 5.60 | 10.27 | 0.04 | 101.90 | 1993.4 | 15.41 | 27.32 | 1.89 | 3.67 | 5.69 | 0.34 |
| Electrical Information Transmission | 4.61 | 4.55 | 9.13 | 11.93 | 0.00 | 171.59 | 1992.5 | 18.86 | 36.77 | 1.69 | 3.42 | 5.55 | 0.16 |
| Electrochemical Battery Energy Storage | 3.88 | 8.99 | 17.70 | 13.27 | 0.02 | 109.58 | 1993.5 | 15.37 | 31.87 | 2.20 | 4.65 | 5.06 | 0.42 |
| Electronic Computation | 6.98 | 3.51 | 7.39 | 15.16 | 0.04 | 192.00 | 1995.4 | 40.50 | 72.02 | 2.22 | 4.35 | 5.78 | 0.60 |
| Flywheel Energy Storage | 3.98 | 18.79 | 33.68 | 16.54 | 0.06 | 15.78 | 1992.1 | 3.16 | 5.19 | 2.31 | 3.80 | 5.50 | 0.40 |
| Fuel Cell Energy Production | 3.89 | 13.82 | 27.55 | 14.19 | 0.02 | 127.61 | 1993.7 | 19.89 | 39.07 | 1.98 | 4.18 | 1.07 | 0.46 |
| Genome Sequencing | 3.32 | 17.40 | 46.28 | 22.56 | 0.33 | 74.10 | 2001.5 | 22.87 | 37.86 | 4.53 | 6.04 | 2.86 | 0.94 |
| Incandescent Artificial Illumination | 2.73 | 12.63 | 23.42 | 9.48 | 0.02 | 21.94 | 1988.0 | 4.10 | 6.24 | 2.06 | 4.24 | 7.10 | 0.18 |
| Integrated Circuit Information Storage | 5.70 | 4.41 | 9.80 | 11.82 | 0.02 | 277.75 | 1996.3 | 46.90 | 90.73 | 2.13 | 5.22 | 3.92 | 0.36 |
| Integrated Circuit Processors | 5.76 | 3.39 | 8.94 | 14.71 | 0.01 | 360.16 | 1996.8 | 62.06 | 123.25 | 2.53 | 7.69 | 4.33 | 0.46 |
| LED Artificial Illumination | 5.88 | 19.21 | 35.18 | 19.13 | 0.02 | 144.16 | 2000.5 | 35.11 | 60.92 | 3.69 | 6.46 | 1.61 | 0.63 |
| Magnet Resonance Imaging (MRI) | 7.67 | 13.71 | 26.71 | 14.82 | 0.05 | 102.33 | 1995.5 | 20.07 | 35.62 | 1.98 | 4.03 | 5.47 | 0.60 |
| Magnetic Information Storage | 4.98 | 4.14 | 8.25 | 11.76 | 0.02 | 146.74 | 1992.2 | 27.17 | 49.54 | 1.96 | 3.88 | 5.50 | 0.29 |
| Milling Machines | 3.05 | 16.50 | 32.18 | 13.42 | 0.03 | 43.22 | 1992.7 | 6.93 | 13.33 | 2.22 | 5.27 | 6.63 | 0.21 |
| Optical Information Storage | 4.58 | 6.32 | 13.99 | 11.91 | 0.01 | 155.18 | 1993.2 | 26.96 | 48.94 | 2.20 | 5.19 | 4.96 | 0.32 |
| Optical Information Transmission | 5.40 | 4.54 | 9.94 | 13.98 | 0.02 | 176.91 | 1992.6 | 27.63 | 49.13 | 1.62 | 4.35 | 4.21 | 0.49 |
| Photolithography | 4.66 | 8.47 | 21.30 | 13.82 | 0.03 | 165.25 | 1998.9 | 35.96 | 70.03 | 2.66 | 5.23 | 4.25 | 0.48 |
| Solar Photovoltaic Energy Generation | 4.47 | 7.82 | 21.56 | 14.97 | 0.04 | 96.96 | 1991.8 | 9.90 | 18.76 | 2.42 | 8.23 | 4.63 | 0.60 |
| Superconductivity | 3.55 | 12.22 | 37.68 | 9.18 | 0.22 | 37.78 | 1992.3 | 8.51 | 13.09 | 2.63 | 7.86 | 4.96 | 0.78 |
| Wind Turbine Energy Generation | 3.47 | 17.97 | 33.55 | 19.62 | 0.05 | 72.92 | 1990.1 | 8.21 | 15.60 | 2.45 | 4.76 | 3.88 | 0.32 |
| Wireless Information Transmission | 5.74 | 3.64 | 7.62 | 16.33 | 0.01 | 258.08 | 1995.2 | 54.51 | 91.87 | 2.69 | 5.45 | 3.65 | 0.46 |
| Mean | 4.73 | 10.09 | 22.36 | 14.26 | 0.05 | 124.03 | 1994.0 | 21.82 | 39.95 | 2.42 | 5.21 | 4.81 | 0.43 |
| SD | 1.25 | 6.14 | 16.48 | 3.20 | 0.07 | 78.82 | 3.1 | 15.14 | 28.47 | 0.64 | 1.51 | 1.37 | 0.18 |
| Min | 2.73 | 2.22 | 3.86 | 9.18 | 0.00 | 15.78 | 1988.0 | 3.16 | 5.19 | 1.62 | 3.42 | 1.07 | 0.16 |
| Max | 7.67 | 24.86 | 82.00 | 22.56 | 0.33 | 360.16 | 2001.5 | 62.06 | 123.25 | 4.53 | 9.32 | 7.10 | 0.94 |

Calculation of these 13 metrics were done similarly as for the 10 in the paper and the equations used are given by:

We ran a correlation using all of the variables in Table C, as shown in Table D. We note that the strongest correlations are reported in the paper. Although the correlation of k with S8 (top 100 patents cited in first 3 years) is strong (r=0.65), the correlation for the same variable for all patents (variable 10) is even stronger (0.76) as reported in the paper. We also note that S13, a different NPL variable than that reported (fraction of patents in a domain without any non patent literature citations) has a different, yet still non-significant correlation with the k-values.

**Table D Correlation values for all variables for 28 domains**

|  | (1) | (2) | (3) | (4) | (5) | (6) | (7) | (8) | (9) | (10) | (S1) | (S2) | (S3) | (S4) | (S5) | (S6) | (S7) | (S8) | (S9) | (S10) | (S11) | (S12) | (S13) | K-Values |
| --- | --- | --- | --- | --- | --- | --- | --- | --- | --- | --- | --- | --- | --- | --- | --- | --- | --- | --- | --- | --- | --- | --- | --- | --- |
| **(1) Simple Patent Count** | 1. |  |  |  |  |  |  |  |  |  |  |  |  |  |  |  |  |  |  |  |  |  |  |  |
| **(2) Average number of forward citations** | 0.01 | 1. |  |  |  |  |  |  |  |  |  |  |  |  |  |  |  |  |  |  |  |  |  |  |
| **(3) Ratio of patents with cited by over 20** | -0.03 | 0.96 | 1. |  |  |  |  |  |  |  |  |  |  |  |  |  |  |  |  |  |  |  |  |  |
| **(4) NPL Ratio** | -0.1 | -0.25 | -0.24 | 1. |  |  |  |  |  |  |  |  |  |  |  |  |  |  |  |  |  |  |  |  |
| **(5) Average publication year** | 0.19 | 0.11 | 0.09 | 0.51 | 1. |  |  |  |  |  |  |  |  |  |  |  |  |  |  |  |  |  |  |  |
| **(6) Average Age of backward Citation** | -0.18 | -0.37 | -0.22 | -0.14 | -0.23 | 1. |  |  |  |  |  |  |  |  |  |  |  |  |  |  |  |  |  |  |
| **(7) Price Index (3 years)** | 0.29 | -0.37 | -0.48 | 0.55 | 0.51 | -0.52 | 1. |  |  |  |  |  |  |  |  |  |  |  |  |  |  |  |  |  |
| **(8) Ratio of Cites to Own Domains** | 0.55 | -0.03 | -0.04 | -0.39 | -0.2 | -0.28 | 0.13 | 1. |  |  |  |  |  |  |  |  |  |  |  |  |  |  |  |  |
| **(9) Total mean publication date of backward citations** | 0.23 | 0.31 | 0.21 | 0.4 | 0.74 | -0.82 | 0.65 | 0.08 | 1. |  |  |  |  |  |  |  |  |  |  |  |  |  |  |  |
| **(10) Average Cited by within 3 years** | 0.26 | 0.77 | 0.64 | -0.03 | 0.4 | -0.73 | 0.27 | 0.13 | 0.74 | 1. |  |  |  |  |  |  |  |  |  |  |  |  |  |  |
| **(S1) Average Cited by within 5 years** | 0.22 | 0.83 | 0.71 | -0.05 | 0.38 | -0.69 | 0.18 | 0.1 | 0.69 | 0.99 | 1. |  |  |  |  |  |  |  |  |  |  |  |  |  |
| **(S2) Average number of UPCs cited (per patent)** | -0.52 | 0.11 | 0.14 | 0.25 | 0.08 | 0.26 | -0.26 | -0.75 | -0.13 | -0.18 | -0.11 | 1. |  |  |  |  |  |  |  |  |  |  |  |  |
| **(S3) Average number of IPCs cited (per patent)** | -0.43 | 0.25 | 0.27 | 0.35 | 0.12 | 0.09 | -0.21 | -0.64 | 0. | -0.05 | 0.02 | 0.92 | 1. |  |  |  |  |  |  |  |  |  |  |  |
| **(S4) Average number of backward citations** | -0.09 | 0.23 | 0.32 | 0.45 | 0.59 | 0.32 | -0.04 | -0.58 | 0.13 | 0.11 | 0.14 | 0.55 | 0.54 | 1. |  |  |  |  |  |  |  |  |  |  |
| **(S5) Top100 NPL %** | -0.24 | -0.29 | -0.27 | 0.89 | 0.2 | 0. | 0.37 | -0.37 | 0.12 | -0.21 | -0.22 | 0.35 | 0.46 | 0.34 | 1. |  |  |  |  |  |  |  |  |  |
| **(S6) Top100 Average number of forward citations** | 0.82 | 0.15 | 0.07 | -0.04 | 0.5 | -0.44 | 0.45 | 0.52 | 0.6 | 0.53 | 0.48 | -0.61 | -0.53 | -0.05 | -0.33 | 1. |  |  |  |  |  |  |  |  |
| **(S7) Top100 Average publication year** | 0.14 | 0.15 | 0.06 | 0.6 | 0.75 | -0.44 | 0.65 | -0.26 | 0.74 | 0.5 | 0.49 | 0.2 | 0.29 | 0.48 | 0.37 | 0.36 | 1. |  |  |  |  |  |  |  |
| **(S8) Top100 Average Cited by within 3 years** | 0.71 | 0.1 | -0.01 | 0.13 | 0.59 | -0.51 | 0.63 | 0.31 | 0.7 | 0.59 | 0.52 | -0.5 | -0.43 | 0.06 | -0.17 | 0.94 | 0.55 | 1. |  |  |  |  |  |  |
| **(S9) Top100 Average Cited by within 5 years** | 0.75 | 0.1 | -0.01 | 0.1 | 0.57 | -0.49 | 0.61 | 0.35 | 0.67 | 0.57 | 0.51 | -0.51 | -0.44 | 0.03 | -0.2 | 0.96 | 0.54 | 0.99 | 1. |  |  |  |  |  |
| **(S10) Top100 Average number of IPC classifications** | -0.11 | -0.21 | -0.23 | 0.65 | 0.44 | 0.05 | 0.47 | -0.53 | 0.22 | -0.03 | -0.06 | 0.36 | 0.41 | 0.55 | 0.61 | -0.12 | 0.63 | 0.13 | 0.09 | 1. |  |  |  |  |
| **(S11) Top100 Average number of UPC classifications** | 0.13 | 0.29 | 0.27 | 0.36 | 0.19 | -0.23 | 0.02 | -0.26 | 0.27 | 0.17 | 0.2 | 0.35 | 0.58 | 0.31 | 0.32 | 0.04 | 0.37 | 0.1 | 0.1 | 0.42 | 1. |  |  |  |
| **(S12) Average Age of Forward Citation** | -0.06 | 0.05 | 0.02 | -0.48 | -0.84 | 0.24 | -0.45 | 0.27 | -0.66 | -0.2 | -0.17 | -0.25 | -0.22 | -0.44 | -0.2 | -0.32 | -0.59 | -0.4 | -0.39 | -0.37 | -0.25 | 1. |  |  |
| **(S13) NPL >0** | -0.11 | 0.14 | 0.13 | 0.88 | 0.53 | -0.36 | 0.42 | -0.45 | 0.56 | 0.3 | 0.29 | 0.32 | 0.44 | 0.48 | 0.72 | 0.02 | 0.63 | 0.18 | 0.15 | 0.5 | 0.52 | -0.53 | 1. |  |
| **K-Values** | 0.33 | 0.48 | 0.38 | 0.2 | 0.54 | -0.59 | 0.39 | 0.11 | 0.72 | 0.76 | 0.73 | -0.14 | -0.04 | 0.24 | -0.03 | 0.6 | 0.5 | 0.65 | 0.62 | -0.02 | 0.11 | -0.38 | 0.39 | 1. |

In order to ensure that the results that we found were not simply due to selection bias of the particular 28 domains, we removed half of the domains and re-ran the correlations – we did this 20 times, and the correlation values for our 20 trials are below in Table E. These values were used to calculate the values in table 5 in the paper.

**Table E Domain Robustness Tests for 10 variables for 20 trials (14 domains each)**

| **Formal Dependent Variable** | **Rand1** | **Rand1b** | **Rand2** | **Rand2b** | **Rand3** | **Rand3b** | **Rand4** | **Rand4b** | **Rand5** | **Rand5B** | **Rand6** | **Rand6B** | **Rand7** | **Rand 7B** | **Rand 8** | **Rand 8B** | **Rand 9** | **Rand 9B** | **Rand 10** | **Rand 10B** |
| --- | --- | --- | --- | --- | --- | --- | --- | --- | --- | --- | --- | --- | --- | --- | --- | --- | --- | --- | --- | --- |
| **(1) Simple Patent Count** | *0.23* | *0.4* | *0.38* | *0.27* | *0.25* | *0.44* | *0.56* | *0.24* | *0.38* | *0.54* | *0.4* | *0.38* | *0.43* | *-0.32* | *0.59* | *0.27* | *0.31* | *0.55* | *0.52* | *0.36* |
| **(2) Average number of forward citations** | *0.52* | *0.55* | *0.13* | *0.56* | *0.43* | *0.48* | *0.4* | *0.52* | *0.49* | *0.45* | *0.69* | *0.26* | *0.61* | *0.37* | *0.7* | *0.53* | *0.51* | *0.45* | *0.54* | *0.34* |
| **(3) Ratio of patents with cited by over 20** | *0.4* | *0.47* | *-0.13* | *0.58* | *0.35* | *0.29* | *0.28* | *0.43* | *0.49* | *0.19* | *0.59* | *0.14* | *0.54* | *0.27* | *0.61* | *0.39* | *0.44* | *0.3* | *0.53* | *-0.04* |
| **(4) NPL Ratio** | *0.21* | *0.49* | *0.39* | *0.17* | *0.32* | *0.31* | *0.3* | *0.16* | *0.17* | *0.16* | *0.19* | *0.17* | *0.68* | *0.26* | *0.14* | *0.28* | *0.53* | *0.15* | *0.17* | *0.46* |
| **(5) Average publication year** | *0.65* | *0.46* | *0.62* | *0.7* | *0.29* | *0.75* | *0.52* | *0.57* | *0.36* | *0.76* | *0.61* | *0.44* | *0.49* | *0.72* | *0.53* | *0.54* | *0.49* | *0.61* | *0.48* | *0.65* |
| **(6) Average Age of Citation** | *-0.62* | *-0.55* | *-0.7* | *-0.41* | *-0.6* | *-0.53* | *-0.63* | *-0.57* | *-0.57* | *-0.61* | *-0.71* | *-0.43* | *-0.65* | *-0.5* | *-0.49* | *-0.73* | *-0.76* | *-0.4* | *-0.55* | *-0.65* |
| **(7) Price Index (3 years)** | *0.28* | *0.61* | *0.68* | *0.04* | *0.24* | *0.58* | *0.58* | *0.24* | *0.17* | *0.6* | *0.37* | *0.37* | *0.66* | *0.26* | *0.46* | *0.36* | *0.45* | *0.36* | *0.25* | *0.63* |
| **(8) Ratio of Cites to Own Domains** | *-0.49* | *0.43* | *0.14* | *-0.04* | *0.* | *0.01* | *0.33* | *-0.14* | *0.19* | *0.12* | *0.35* | *-0.09* | *0.43* | *-0.46* | *0.11* | *0.11* | *0.2* | *-0.14* | *0.28* | *-0.09* |
| **(9) Total mean publication date of backward citations** | *0.84* | *0.6* | *0.81* | *0.67* | *0.61* | *0.81* | *0.72* | *0.74* | *0.61* | *0.82* | *0.84* | *0.62* | *0.71* | *0.79* | *0.64* | *0.84* | *0.73* | *0.71* | *0.65* | *0.84* |
| **(10) Average Cited by within 3 years** | *0.84* | *0.68* | *0.76* | *0.72* | *0.7* | *0.79* | *0.76* | *0.76* | *0.7* | *0.82* | *0.94* | *0.57* | *0.76* | *0.71* | *0.79* | *0.8* | *0.79* | *0.73* | *0.77* | *0.76* |

An important issue is the ability of the correlations to work in the future not just in the past. A second robustness test also tests the predictive capability of the correlations by testing how sensitive the patent metrics correlations were to variations in time. In order to do this, the patent metrics were analyzed for only patents from a variety of time frames that were less than the total time frame. The time frames were analyzed to see how far back from 2013 they could be analyzed and still find similar correlations as the patent metrics show during the entire time frame (1976-2013). The results of these time based tests for the two robust, strong signals that combine to form our strongest multiple regression are shown in table F.

**Table F Time Robustness Analysis for 2 Patent metrics showing Pearson correlation and p-values**

|  | **All Data** | **2001** | **1991** | **1981** |
| --- | --- | --- | --- | --- |
|  | **1976-2013** | **(26 years)** | **(16 years)** | **(6 years)** |
|  | **(38 years)** |  |  |  |
| **Cited by Within 3 Years** | Cp = 0.76 | 0.72 | 0.52 | 0.3 |
|  | *2.6*10^-6^* | *.00002* | *.004* | *0.12* |
| **Average Publication Year** | Cp = 0.54 | 0.53 | -0.07 | -0.18 |
|  | *.003* | *.003* | *0.71* | *0.36* |

The two patent metrics show very similar correlations for 1976-2001 than they do for the entire data set of 1976-2013 and the p values for both correlations are still superb with the more restricted patent data. This indicates that the metrics have the potential for predictive capabilities of 12 years or more into the future. When the patent metrics were analyzed for patents before 1991, the correlation value of the cited by within 3 years metric shows some drop-off (from 0.72 to 0.52) and the average publication year shows a significant drop-off and has a very high p-value which indicates that the signal is not at all reliable for 22 years. The decrease in reliability of the correlation back to 1991 is at least partly due to the fact that many of the domains were very young (and in two cases non-existent) at that point in time and thus there were very few patents to analyze. The data from the 1976-1981 timeframe shows that both patent metrics are non-signals for the short 6-years timeframe. Ultimately the two strongest and most robust patent metrics are robust to time up to 12 years prior to the experiment reported in detail here, showing a promising amount of predictive capability.

In order to test the effects of noise on the R^2^ values of the models, we used Model D and tested to see the variation in R2 given a certain noise in the k-values even if we had a perfect model. In order to do this we used model D to create a mean k-value for each of the 28 domains, and assumed a normal distribution with a standard deviation of 0.25 times the mean. From here we randomly selected a set of 28 k-values from those distributions and compared the R^2^ between these 28 values and the 28 values calculated from Model D (our ideal model in this case). This was repeated 10,000 times and the average R^2^ between the noisy k-values and the ideal k-values was 0.809. The calculated K-values and standard deviations are shown below in Table G. An example set of 28 k-values from one of the randomized runs is also shown.

**Table G Testing the effect of noisy k-values on model R^2^**

| **FormalDependent Variable** | **Calculated K From Model D** | **Stdev** | **Randomly selected set of k-values** |
| --- | --- | --- | --- |
| 3D-Printing (industrial stereolithography) | 38.4% | 9.6% | 41.5% |
| Aircraft Transport | 5.5% | 1.4% | 5.9% |
| Camera Sensitivity | 30.2% | 7.5% | 30.8% |
| Capacitor Energy Storage | 21.0% | 5.3% | 20.1% |
| Combustion Engines | 14.2% | 3.5% | 12.1% |
| Computed Tomography (CT) | 27.0% | 6.8% | 19.6% |
| Electric Motors | 11.5% | 2.9% | 14.0% |
| Electrical Energy Transmission | 23.3% | 5.8% | 25.1% |
| Electrical Information Transmission | 21.3% | 5.3% | 17.7% |
| Electrochemical Battery Energy Storage | 13.4% | 3.4% | 11.9% |
| Electronic Computation | 46.0% | 11.5% | 15.4% |
| Flywheel Energy Storage | 8.2% | 2.0% | 4.3% |
| Fuel Cell Energy Production | 22.3% | 5.6% | 25.3% |
| Genome Sequencing | 20.9% | 5.2% | 12.3% |
| Incandescent Artificial Illumination | -2.9% | 0.7% | -2.2% |
| Integrated Circuit Information Storage | 34.0% | 8.5% | 40.6% |
| Integrated Circuit Processors | 36.0% | 9.0% | 38.3% |
| LED Artificial Illumination | 43.6% | 10.9% | 35.0% |
| Magnet Resonance Imaging (MRI) | 46.9% | 11.7% | 44.4% |
| Magnetic Information Storage | 23.2% | 5.8% | 20.4% |
| Milling Machines | 0.7% | 0.2% | 0.8% |
| Optical Information Storage | 22.0% | 5.5% | 18.4% |
| Optical Information Transmission | 31.3% | 7.8% | 39.5% |
| Photolithography | 26.2% | 6.6% | 23.0% |
| Solar Photovoltaic Energy Generation | 16.5% | 4.1% | 17.1% |
| Superconductivity | 9.7% | 2.4% | 11.0% |
| Wind Turbine Energy Generation | 15.1% | 3.8% | 8.0% |
| Wireless Information Transmission | 39.1% | 9.8% | 49.0% |
